# SPAG6基因沉默和地西他滨对SKM-1细胞凋亡和PTEN甲基化的影响

**DOI:** 10.3760/cma.j.issn.0253-2727.2021.12.007

**Published:** 2021-12

**Authors:** 洁 罗, 佼 牟, 林 刘

**Affiliations:** 1 重庆医科大学附属第一医院血液科 400016 Department of Hematology, The First Affiliated Hospital of Chongqing Medical University, Chongqing 400016, China; 2 重庆医科大学实验研究中心400016 Experimental Research Center, Chongqing Medical University, Chongqing 400016, China

**Keywords:** 骨髓增生异常综合征, 精子相关抗原6, PTEN基因, 地西他滨, Myelodysplastic syndrome, Sperm associated antigen 6, PTEN gene, Decitabine

## Abstract

**目的:**

探讨SPAG6基因沉默和地西他滨在体内外对SKM-1细胞凋亡和PTEN启动子甲基化的作用。

**方法:**

慢病毒载体转染SKM-1细胞沉默SPAG6基因的表达，地西他滨处理细胞后CCK-8检测细胞存活率，流式细胞术检测细胞凋亡，Western blot和甲基化特异性PCR检测PTEN的蛋白表达和甲基化情况，并构建非肥胖糖尿病/重度联合免疫缺陷（NOD/SCID）小鼠异体移植瘤模型，TUNEL法观察肿瘤组织凋亡，免疫组化检测PTEN在组织中表达情况。

**结果:**

慢病毒转染后SPAG6干扰组SPAG6基因被成功沉默；CCK-8检测结果提示地西他滨处理能够降低SKM-1细胞的存活率，同时流式细胞术检测显示地西他滨处理组的细胞凋亡率［（17.35±3.37）％］高于未处理组［（5.09±2.06）％］，且SPAG6基因沉默联合地西他滨处理组的凋亡率最高［（36.34±4.00）％］；地西他滨处理后DNMT1的表达降低而PTEN表达升高，同时PTEN启动子甲基化程度降低，而经地西他滨处理后的SPAG6干扰组的PTEN去甲基化引起的蛋白表达升高效果最明显；NOD/SCID小鼠异体瘤移植模型中地西他滨组的肿瘤体积明显小于生理盐水组，而地西他滨处理SPAG6干扰组的小鼠肿瘤体积最小，且经TUNEL检测发现其凋亡程度最高（阳性比值为3.57±0.48）。

**结论:**

SPAG6基因沉默在SKM-1细胞中能增强地西他滨诱导的凋亡和PTEN去甲基化作用，并且在NOD/SCID异体瘤移植模型小鼠中能够增强地西他滨的抗肿瘤特性。

## Abstract (en)

**Objective:**

To investigate the effects of SPAG6 silencing and decitabine on apoptosis and phosphatase and tensin homolog (PTEN) methylation in SKM-1 cells in vitro and in vivo.

**Methods:**

SKM-1 cells were transfected with a lentiviral vector to silence the expression of SPAG6. Cell survival rate was detected by CCK8 after treatment with decitabine, and cell apoptosis was detected by flow cytometry. Protein expression and methylation of PTEN were detected using Western blot and merozoite surface protein (MSP). An non-obese diabetic/severe combined inmunodeficiency disease (NOD/SCID) mice xenograft tumor model was established, and the apoptosis and PTEN expression of tumor tissue were observed through terminal deoxynucleotidyl transferase dUTP nick end labeling (TUNEL) and immunohistochemistry (IHC), respectively.

**Results:**

After lentivirus transfection, SPAG6 in the interference group was silenced successfully. CCK8 results indicated that the cell survival rate of SKM-1 cells treated with decitabine decreased. Flow cytometry showed that the apoptosis rate of cells treated with decitabine [(17.35±3.37)%] was higher than that of the untreated group (5.09%±2.06%) and the apoptosis rate of the SPAG6 silencing combined with the decitabine treatment group was the highest [(36.34±4.00)%]. After treatment with decitabine, the expression of DNMT1 decreased, while the expression of PTEN increased, and the promoter methylation degree of PTEN also decreased. Moreover, the increased protein level caused by PTEN demethylation was the most obvious in the SPAG6 in the interference shRNA group treated with decitabine. In NOD/SCID mice, the tumor volume of the decitabine group was significantly smaller than that of the placebo group, and the tumor volume of the SPAG6 silencing combined with the decitabine treatment group was the smallest. Additionally, the apoptosis rate was the highest (the positive ratio was 3.57±0.48).

**Conclusion:**

SPAG6 silencing may enhance the apoptosis level and the effect of PTEN demethylation in SKM-1 cells and enhance the antitumor effect of decitabine in the NOD/SCID xenograft mouse model.

骨髓增生异常综合征（MDS）是一组起源于多能造血干/祖细胞的恶性克隆性血液疾病，老年人群多见，以无效造血、难治性血细胞减少及高风险向急性髓系白血病（AML）转化为特征[1]。其治疗方案包括支持治疗、免疫治疗、去甲基化治疗以及造血干细胞移植等[2]–[3]，其中地西他滨常被用于MDS的临床治疗，但治疗仍存在局限性[4]–[5]。精子相关抗原6（SPAG6）是一种新型肿瘤睾丸抗原[6]–[8]，课题组前期的研究中证实SPAG6可能和MDS细胞株SKM-1细胞的增殖、凋亡和自噬等活动相关，并参与PI3K/AKT、mTOR等多个通路的调控[9]–[12]，沉默SPAG6基因可下调SKM-1细胞中甲基转移酶DNMT1的表达[13]。本研究中，我们通过构建慢病毒载体沉默SPAG6基因，并使用地西他滨处理细胞，探究SPAG6基因沉默与地西他滨处理对SKM-1细胞的凋亡和对PTEN的去甲基化作用，以期探索分子靶向和去甲基化药物联合作用治疗MDS的可能。

## 材料与方法

1. 主要试剂：CCK-8试剂购于日本Dojindo公司，DNA提取试剂盒购于天根生化科技（北京）有限公司，亚硫酸氢盐处理试剂盒购于美国Zymo research公司，TUNEL试剂盒、GAPDH抗体购于上海碧云天生物技术有限公司，地西他滨购于美国MedChem Express公司，SPAG6抗体购于美国Sigma公司，PTEN抗体购于美国Cell Signaling公司，DNMT1抗体购于美国Abcam公司。

2. 细胞株培养：SKM-1细胞由课题组液氮冻存，使用RPMI培养基（含10％的FBS）培养；人肾上皮细胞株293T细胞使用DMEM培养基（含10％FBS），在37°C，5％CO_2_的条件下培养。

3. SKM-1细胞转染：慢病毒载体的序列和构建参考课题组前期研究[14]。收集呈对数生长的SKM-1细胞，以细胞密度5×10^4^个/ml种板于24孔板中，每孔500 µl，并加入终浓度5 µg/ml的聚凝胺助染，37 °C孵育2 h后，按照转染复数（MOI）＝100进行转染，转染24 h后更换新鲜培养基终止转染，并于转染后3 d荧光显微镜下观察细胞。

4. 蛋白提取和Western blot检测蛋白表达：收集转染5 d后的细胞，加入RIPA冰上裂解细胞后，4 °C、12 000×*g*离心15 min并收集上清液，通过BCA试剂盒测蛋白浓度。总蛋白40 µg用10％的SDS-PAGE凝胶电泳分离后转移到PVDF膜上，然后5％的脱脂牛奶室温摇床上封闭2 h，4 °C一抗孵育过夜，次日用山羊抗体二抗室温孵育1 h，最后ECL化学发光法显色检测蛋白表达信号，实验重复3次。

5. CCK-8检测细胞增殖：收集阴性对照（NC）-shRNA和SPAG6-shRNA组SKM-1细胞接种于96孔板中，每孔5×10^3^个细胞，并加入100 µl的培养基，分别用0、0.1、0.5、1、2、4、6、8和10 µmol/L终浓度的地西他滨处理细胞，72 h后向每孔中加入10 µl的CCK-8试剂，37°C孵箱中孵育2 h，酶标仪检测450 nm处的吸光度（*A*）值，并计算细胞存活率，每次实验设3个复孔，实验重复3次。

6. 细胞DNA提取和甲基化特异性PCR：收集细胞，根据DNA提取试剂盒说明书提取细胞DNA，通过EZ DNA甲基化试剂盒进行亚硫酸氢盐转化。扩增方案：95 °C预变性10 min；95 °C变性30 s，60 °C退火30 s，72 °C延伸30 s，40个循环；72 °C延长7 min。引物序列：未甲基化上游引物：5′-TATTAGTTTGGGGATTTTTTTTTTG-3′，下游引物：5′-CCCAA-CCCTTCCTACACCACA-3′；甲基化上游引物：5′-G-TTTGGGGATTTTTTTTTCGC-3′，下游引物：5′-AA-CCCTTCCTACGCCGCG-3′。扩增产物经2％琼脂糖凝胶电泳成像。

7. Annexin Ⅴ/PI双染法检测细胞凋亡率：2 µmol/L地西他滨或者生理盐水处理72 h后收集细胞，400 µl的PBS重悬，并分别加入5 µl PI和5 µl Annexin Ⅴ，充分混匀后室温避光反应10 min后上流式细胞仪检测，实验重复3次。

8. 小鼠异体移植瘤的构建：北京华阜康公司购买4～5周龄的雄性非肥胖糖尿病/重度联合免疫缺陷（NOD/SCID）小鼠，每只重18～25 g，饲养于重庆医科大学实验动物中心。随机将小鼠分为4组，每组4只，收集NC-shRNA和SPAG6-shRNA组SKM-1细胞，各组小鼠皮下注射1×10^7^个SKM-1细胞。14 d后，治疗组小鼠腹腔注射地西他滨1 mg·kg^−1^·d^−1^，安慰剂组小鼠腹腔注射等体积生理盐水，连续给药5 d，每周3次观察小鼠体重及肿瘤生长情况。药物治疗后14 d或者小鼠肿瘤直径大于2 cm或小鼠一般状况差时处死小鼠，并分离肿瘤组织。所有动物实验操作均经重庆医科大学动物护理与使用委员会批准。

9. TUNEL检测细胞凋亡：肿瘤组织切片后二甲苯脱蜡5 min，乙醇100％、90％、80％、70％浓度梯度水合，每次2 min，PBS冲洗5 min，加入蛋白酶K工作液，37 °C作用20 min后PBS洗涤，3％ H_2_O_2_作用10 min，PBS洗涤，向组织样本上滴加50 µl生物标记液，37 °C作用1 h后终止，室温孵育HRP工作液后DAB显色剂显色。

10. 免疫组化分析：肿瘤组织切片经脱蜡水化后进行抗原修复，并向切片上滴加适量的内源性过氧化物酶，室温孵育10 min，再滴加山羊血清封闭20 min后滴加一抗稀释液，4 °C孵育过夜，之后滴加适量生物素标记山羊抗兔IgG聚合物，室温孵育15 min，再滴加辣根酶标记链霉卵白素工作液，室温孵育15 min后DAB显色液染色，苏木素复染，二甲苯透明2次，中性树胶封片。

11. 统计学处理：使用SPSS 22.0和Graphpad 8.0进行数据分析，所有数据以均数±标准差表示，两组之间比较采用*t*检验，多组之间的比较采用单因素方差分析，*P*<0.05为差异有统计学意义。

## 结果

1. 慢病毒载体的转染效率：慢病毒转染效率如图1所示，NC-shRNA和SPAG6-shRNA转染SKM-1细胞后荧光显微镜下可见细胞中有大量的绿色荧光信号的表达，表明NC-shRNA和SPAG6-shRNA均成功转染SKM-1细胞。

**图1:**
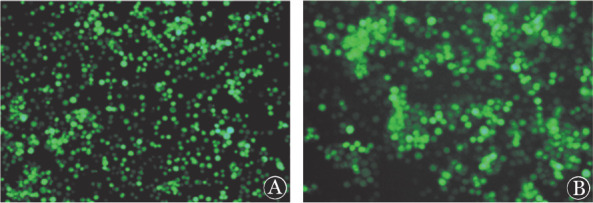
慢病毒转染SKM-1细胞后荧光显微镜观察荧光信号 A：阴性对照组；B：SPAG6干扰组

2. 慢病毒干扰对SPAG6和PTEN蛋白表达的影响：Western blot检测SPAG6蛋白表达情况，较阴性对照组，SPAG6干扰组SPAG6蛋白表达下调、PTEN蛋白表达上调（图2）。

**图2:**
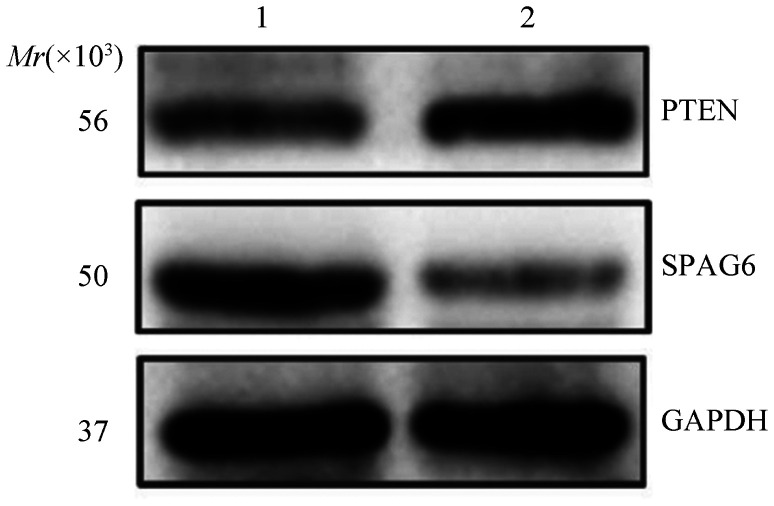
Western blot检测SPAG6-shRNA转染后SKM-1细胞SPAG6和PTEN的表达情况 1：阴性对照组；2：SPAG6干扰组

3. 地西他滨处理SKM-1细胞后细胞活力检测：不同终浓度地西他滨处理阴性对照组和SPAG6干扰组SKM-1细胞，CCK-8检测细胞存活率。结果显示，各浓度地西他滨均可降低细胞存活率，其中SPAG6干扰组的存活率均低于阴性对照组，且细胞存活率随着药物浓度增加而降低（图3）。

**图3:**
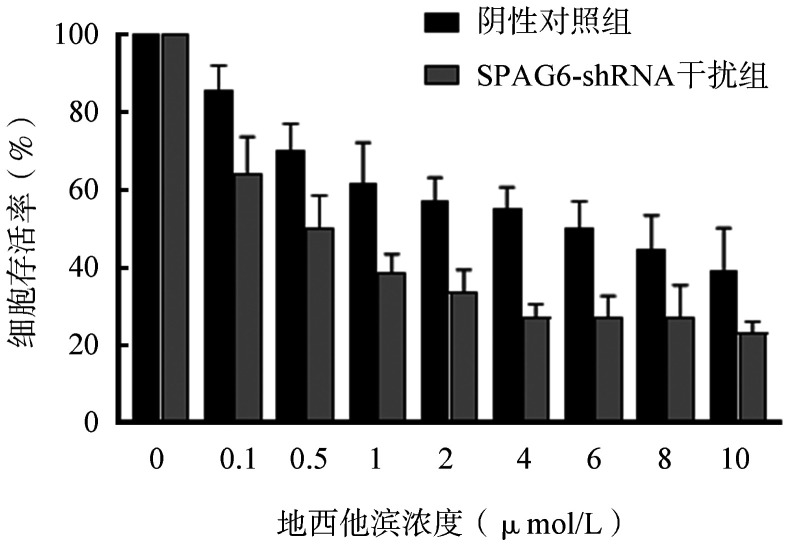
不同浓度地西他滨处理SKM-1细胞72 h后CCK8检测细胞活力

4. SPAG6沉默和地西他滨处理后SKM-1细胞PTEN的表达和甲基化：2 µmol/L地西他滨处理SKM-1细胞72 h，Western blot检测示DNMT1表达下调，且PTEN的表达升高；同时甲基化特异性PCR结果示，与地西他滨未处理组相比，地西他滨处理后PTEN的启动子甲基化程度降低，SPAG6沉默联合地西他滨处理对PTEN的去甲基化最强、表达上调最明显（图4）。

5. SPAG6沉默和地西他滨处理后细胞凋亡率的变化：2 µmol/L的地西他滨处理阴性对照组和SPAG6干扰组细胞，72 h后流式细胞术检测凋亡率。结果显示，阴性对照组凋亡率为（5.09±2.06）％，阴性对照+地西他滨组凋亡率为（17.35±3.37）％，SPAG6组凋亡率为（16.57±2.85）％，SPAG6+地西他滨组凋亡率为（36.34±4.00）％，提示SPAG6基因沉默和地西他滨处理均可引起SKM-1细胞的凋亡增加，而SPAG6基因沉默和地西他滨处理对凋亡有协同作用（图5）。

**图4:**
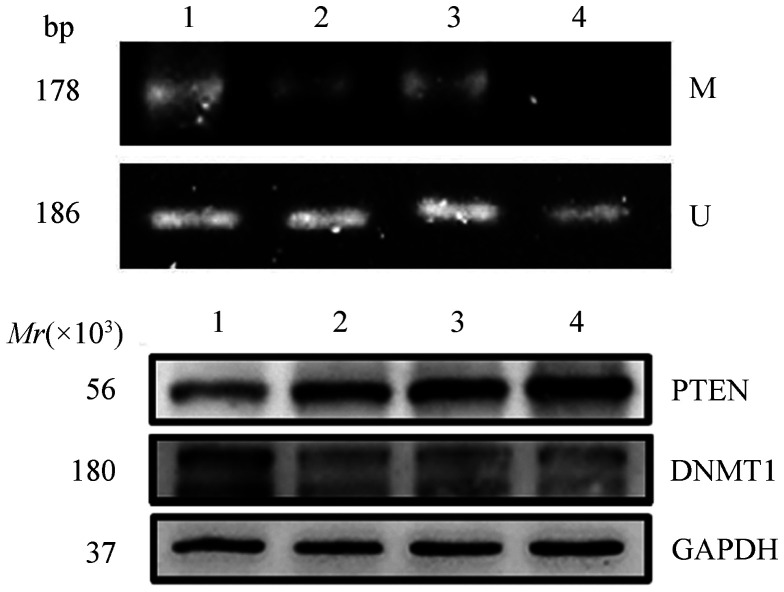
地西他滨处理SKM-1细胞72 h后PTEN启动子甲基化（A）以及PTEN和DNMT1蛋白（B)的表达情况 1：阴性对照组；2：地西他滨处理阴性对照组；3：SPAG6干扰组；4：地西他滨处理SPAG6干扰组

**图5:**
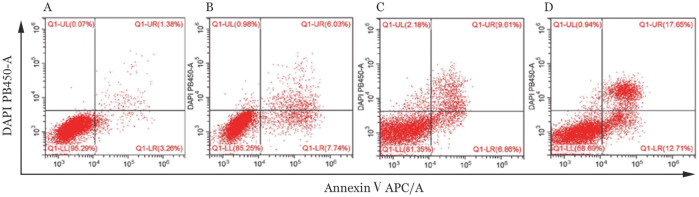
流式细胞学检测地西他滨处理后细胞的凋亡率 A：阴性对照组；B：地西他滨处理阴性对照组；C：SPAG6干扰组；D：地西他滨处理SPAG6干扰组

6. SPAG6基因沉默和地西他滨处理对小鼠异体移植瘤的影响：构建NOD/SCID小鼠的异体移植瘤模型，结果如图6所示，地西他滨处理组的小鼠肿瘤体积小于生理盐水组，SPAG6干扰组的小鼠肿瘤体积较阴性对照组的小鼠体积更小，其中地西他滨处理后的SPAG6干扰组小鼠肿瘤体积缩小最为明显，提示地西他滨在小鼠体内有抗肿瘤特性。

**图6:**
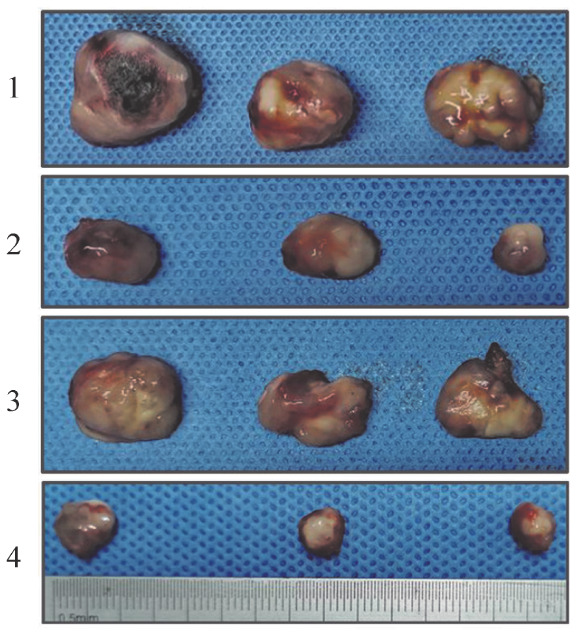
不同处理组小鼠异体移植瘤组织大小

7. TUNEL检测小鼠移植瘤的凋亡情况：TUNEL法检测组织原位细胞的凋亡率，地西他滨处理组小鼠的肿瘤组织阳性区域面积高于阴性对照组，同理SPAG6干扰组肿瘤组织阳性区域面积也高于阴性对照组（图7）。同时，地西他滨处理后的SPAG6干扰组凋亡率最高，与体外研究结果一致，提示地西他滨可以诱导SKM-1细胞体内体外的凋亡，而SPGA6能够增强地西他滨的促凋亡作用。

**图7:**
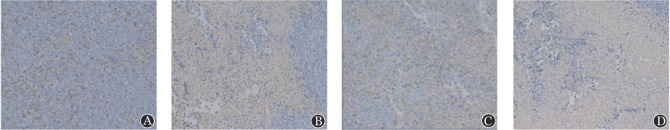
不同处理组TUNEL检测肿瘤组织原位细胞凋亡情况（×200） A：阴性对照组；B：阴性对照组加地西他滨处理组；C：SPAG6干扰组加生理盐水组；D：SPAG6干扰组加地西他滨处理组

8. 小鼠移植瘤组织的PTEN的表达：Western blot和免疫组化检测小鼠移植瘤组织中PTEN的DNMT1的表达情况，结果提示与阴性对照组相比，地西他滨处理组的DNMT1表达降低而PTEN表达升高，同时SPAG6干扰组较阴性对照组DNMT1表达水平更低，PTEN表达水平更高，SPAG6沉默联合地西他滨处理组的DNMT1降低和PTEN升高最明显（图8、图9）。

**图8:**
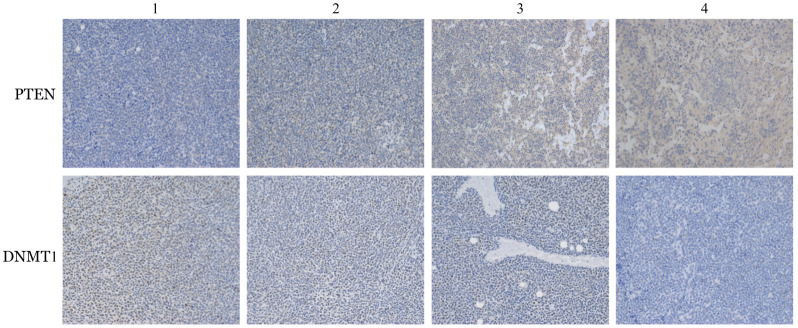
免疫组化检测不同处理组异体移植瘤模型NOD/SCID小鼠肿瘤组织中PTEN和DNMT1蛋白的表达情况 1：阴性对照组；2：地西他滨处理的阴性对照组；3：SPAG6干扰组；4：地西他滨处理的SPAG6干扰组

**图9:**
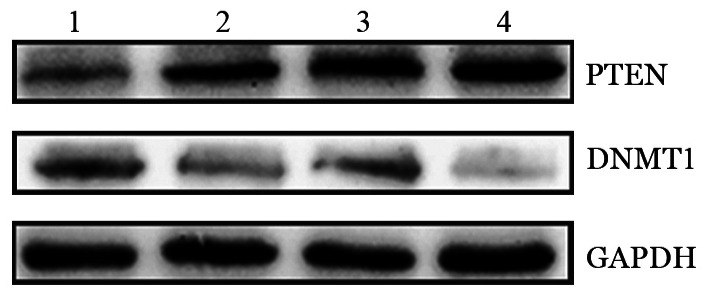
Western blot检测不同处理组异体移植瘤模型NOD/SCID小鼠肿瘤组织中PTEN和DNMT1蛋白的表达情况 1：阴性对照组；2：地西他滨处理的阴性对照组；3：SPAG6干扰组；4：地西他滨处理的SPAG6干扰组

## 讨论

MDS是一种起源于多能造血干细胞的恶性克隆性血液疾病，目前的研究认为其发病机制可能和骨髓微环境、体细胞突变、单倍体不足和表观遗传学变化等多种因素有关[1]。本课题组前期研究发现，SPAG6基因在MDS/AML中高表达[10]，且可能通过影响抑癌基因PTEN的表达来调控PI3K/AKT通路，但具体SPAG6影响PTEN表达的机制尚未阐明[13]。在近来的研究中，前列腺癌、乳腺癌、子宫内膜癌、肺癌和其他恶性肿瘤中发现了PTEN启动子高甲基化水平[15]–[19]，并被认为其高甲基化水平可能和甲基转移酶的调控有关[20]–[21]。

表观遗传包括DNA甲基化和组蛋白修饰等，异常的DNA甲基化可作为肿瘤的生物学标志物[21]。地西他滨作为一种DNA去甲基化药物，能够竞争性抑制DNA甲基转移酶的活性，并通过去甲基化作用[22]–[23]激活肿瘤抑制基因，常用于MDS的治疗。除了在血液系统恶性肿瘤中的应用，地西他滨目前也在一些实体肿瘤（包括卵巢癌、前列腺癌和结直肠癌等）中进行临床试验[24]–[26]。在本研究中，地西他滨处理细胞后，DNMT1表达下调，PTEN表达上调，同时PTEN的启动子甲基化程度降低，而SPAG6基因沉默能够增加地西他滨对PTEN去甲基化的作用。

Wu等[27]发现地西他滨可以引起SKM-1细胞凋亡率增加，且检测到凋亡抑制因子Bcl-2表达下调和促凋亡因子Bax表达增高，而韩颖等[28]的研究表明，地西他滨对细胞的增殖杀伤有着浓度依赖的作用，且影响着SKM-1细胞的凋亡和侵袭等生物学功能。在本研究中，流式细胞术检测发现地西他滨处理组的细胞凋亡率均高于未处理组，且SPAG6基因沉默可以增加地西他滨的促凋亡作用。

本课题组前期研究通过皮下成瘤构建SKM-1细胞小鼠异体移植瘤模型，结果提示SPAG6基因沉默能够使小鼠异体移植瘤体积缩小[13],[29]。本研究结果与前期研究一致。此外，本研究我们还发现地西他滨处理组的小鼠肿瘤体积较生理盐水组体积更小，且TUNEL检测地西他滨处理组的凋亡程度高于组，提示地西他滨在小鼠体内有抗肿瘤的作用，而地西他滨作用于SPAG6干扰组后小鼠的移植瘤体积缩减最多，提示SPAG6基因沉默可能增强地西他滨的体内抗肿瘤作用。

综上所述，本研究结果提示地西他滨有诱导SKM-1细胞凋亡和PTEN去甲基化的作用，且在小鼠移植瘤模型中表现出抗肿瘤生长的作用，而SPAG6基因沉默后能够增强地西他滨的体内外作用，因此SPAG6靶向沉默联合地西他滨或许能为MDS治疗提供新的思路。
